# Cultural Adaptation and Psychometric Properties of the Spanish Version of the Gay Affirmative Practice Scale (GAP-ES)

**DOI:** 10.3390/healthcare12222258

**Published:** 2024-11-13

**Authors:** Piotr Karniej, Anthony Dissen, Raúl Juárez-Vela, Antonio Martinez Sabater, Pablo del Pozo-Herce, Vicente Gea-Caballero, Emmanuel Echaniz-Serrano, Michał Czapla

**Affiliations:** 1 Faculty of Finance and Management, WSB MERITO University in Wroclaw, 53-609 Wrocław, Poland; 2 Group of Research in Care (GRUPAC), Faculty of Health Sciences, University of La Rioja, 26006 Logroño, Spain; raul.juarez@unirioja.es (R.J.-V.); antonio.martinez-sabater@uv.es (A.M.S.); michal.czapla@umw.edu.pl (M.C.); 3 School of Health Sciences, Stockton University, Galloway, NJ 08205, USA; anthony.dissen@stockton.edu; 4 Faculty of Nursing, Nursing Care and Education Research Group (GRIECE), GIUV2019-456, Nursing Department, University of Valencia, 46010 Valencia, Spain; 5 Care Research Group (INCLIVA), Hospital Clínico Universitario de Valencia, 46010 Valencia, Spain; 6 Research Group on Innovation in Health Care and Nursing Education (INcUidE), UNIE Universidad, 28040 Madrid, Spain; pablo.delpozo@universidadunie.com; 7 Faculty of Health Sciences, Research Group Community Health and Care, International University of Valencia, 46002 Valencia, Spain; vagea@universidadviu.com; 8 Sapienf (B53_23R) Research Group, Department of Physiatry and Nursing, Faculty of Health Sciences, University of Zaragoza, 50009 Zaragoza, Spain; eechaniz@unizar.es; 9 Department of Emergency Medical Service, Faculty of Nursing and Midwifery, Wrocław Medical University, 51-616 Wrocław, Poland; 10 Institute of Heart Diseases, University Hospital, 50-556 Wroclaw, Poland

**Keywords:** gender minorities, gay people, gay affirmative, acceptability of healthcare, gay, LGBT health care

## Abstract

Background/Objectives: Lesbian, gay, bisexual, and transgender (LGBT) individuals often face discrimination in healthcare settings, resulting in health disparities. Evaluating healthcare professionals’ affirmative practices is essential for promoting inclusive care and addressing these disparities. The aim of this study was to assess the psychometric properties of the Spanish version of the Gay Affirmative Practice Scale (GAP-ES), which measures healthcare professionals’ affirmative practices towards gay individuals. Methods: Before assessing its psychometric properties, the original Gay Affirmative Practice Scale (GAP) was translated and culturally adapted from English to Spanish. Following the translation, the psychometric properties were tested on a sample of 236 healthcare professionals. The internal consistency of the questionnaire was measured using Cronbach’s alpha and the discriminatory power index. Factor structure was evaluated with Confirmatory Factor Analysis (CFA) using the Diagonally Weighted Least Squares method. Results: The sample consisted of 152 female (64.41%) and 84 male (35.59%) participants, with 58.05% identifying as heterosexual, 28.81% as homosexual, and 13.14% as bisexual. The internal consistency of the GAP-ES was strong, with Cronbach’s alpha values of 0.915 for the Beliefs subscale and 0.902 for the Behaviors subscale. The McDonald’s Omega coefficient was 0.942, indicating high reliability. CFA confirmed a two-factor structure with satisfactory fit indices (CFI = 0.999, RMSEA = 0.071). Conclusions: The GAP-ES demonstrates strong internal consistency and a stable factor structure. It is a reliable tool for evaluating affirmative practices toward LGBT patients in Spanish-speaking healthcare contexts, supporting improved care for this population. The integration of the GAP-ES into clinical practice and training programs may support the enhancement of cultural competence among healthcare professionals, contributing to the reduction of health disparities for LGBT patients in Spanish-speaking settings.

## 1. Introduction

The lesbian, gay, bisexual, and transgender (LGBT) community represents a diverse population that has historically faced discrimination, prejudice, marginalization, and abuse, which has led to significant health disparities, including disproportionate physical and mental health outcomes [1]. Despite improvements in societal attitudes toward members of the LGBT community over recent decades, these health disparities persist [2].

LGBT individuals often avoid engaging with healthcare services or distrust medical professionals due to previous or anticipated experiences of stigmatization and discrimination during medical encounters, including outright denial of care [2,3]. These disparities, linked to social and structural inequities, directly contribute to negative health outcomes for LGBT patients, affecting areas such as sexual and reproductive health, mental health, and outcomes related to cardiovascular diseases and cancers [1,4].

A lack of clinically and culturally competent healthcare providers remains a significant concern for members of the LGBT community, as highlighted by the National Academies of Sciences, Engineering, and Medicine [4,5]. In response, national organizations have developed protocols and guides to assist medical institutions and professionals in providing more affirming care [4,6]. Despite these initiatives, there is a widespread shortage of training focused on equipping healthcare providers with the clinical and cultural competencies necessary to meet the often unmet and unique health needs of LGBT patients [6,7].

Healthcare professionals frequently acknowledge that they lack sufficient training in providing care to LGBT patients [4,8,9]. Studies indicate that the lack of specialized training is a primary barrier to delivering appropriate care [4,6,10]. Moreover, many medical professionals express a desire for additional training to better understand and address the needs of this population, with most agreeing that such training should be mandatory [4,11].

In Spain, despite advances in LGBT rights, challenges remain in ensuring appropriate and affirming healthcare for this population. Research indicates that members of the LGBT community in Spain experience discrimination, violence, and inadequate medical care, which negatively affects their physical and mental health [6,12,13,14]. There is a need to enhance cultural competencies among Spanish healthcare professionals to effectively respond to the diverse needs of LGBT individuals and to prevent further stigmatization and health disparities resulting from a lack of competence [4,6]. While discrimination and inadequate care for LGBT individuals in Spain have been documented, research has yet to thoroughly explore healthcare providers’ ability to implement affirming practices and their preparedness to engage with LGBT patients in a supportive manner [15]. This gap highlights the need for culturally adapted tools, which can provide a meaningful assessment of these practices in Spanish-speaking healthcare contexts and guide improvements in LGBT patient care.

Cultural adaptation and validation of tools measuring affirmative practices toward homosexual individuals are crucial for improving the quality of healthcare [16]. The Gay Affirmative Practice Scale (GAP) is one of the tools available globally for assessing acceptance or affirmation toward the LGBT population, focusing on evaluating professionals’ practices and attitudes [16,17]. The lack of a culturally adapted, psychometrically validated tool in Spanish that evaluates healthcare providers’ affirmative practices limits both research and the development of targeted training interventions. The absence of a Spanish version of this instrument hinders the assessment and training of healthcare providers in Spain regarding affirmative practices toward homosexual individuals.

Therefore, the purpose of this study is to evaluate the psychometric properties of the Spanish-language version of The Gay Affirmative Practice Scale (GAP-ES). Adapting this tool will contribute to a better understanding and improvement of medical practices toward members of the LGBT community in Spain, enabling professionals to more effectively meet the unique needs of gay patients and reduce health disparities arising from a lack of cultural competence.

## 2. Materials and Methods

### 2.1. Study Design and Participants

A cross-sectional study was conducted between April and August 2024 to evaluate the psychometric properties of the Spanish version of the Gay Affirmative Practice Scale (GAP-ES) (Supplementary Materials). A total of 236 participants were recruited using convenience sampling methods through social media platforms, including Instagram and Facebook groups dedicated to healthcare professionals. The sample comprised 84 men and 152 women. Participants’ sexual orientation was reported as heterosexual (137 individuals, 58.05%), homosexual (68 individuals, 28.81%), and bisexual (31 individuals, 13.14%).

Inclusion criteria for the study were being a medical student or a practicing healthcare professional and providing informed consent to participate. Participants were fully informed about the study’s objectives and procedures before completing an anonymous, self-administered questionnaire divided into two sections. The first section collected demographic information such as age, gender, sexual orientation, place of residence, and professional role within the healthcare sector. The second section consisted of the GAP-ES instrument.

Data were collected using an online survey hosted on the Webankieta platform [18]. The platform provided secure data handling and utilized IP filtering to prevent multiple submissions from the same participant, ensuring data integrity. Participants accessed the survey through a link distributed on social media and professional networks. Upon accessing the survey, participants were presented with an informed consent form outlining the study’s purpose, procedures, confidentiality measures, and the voluntary nature of participation. Only after providing consent could participants proceed to complete the questionnaire.

According to sample size recommendations for psychometric evaluations, a minimum of 5 to 10 participants per item is suggested [19]. Given that the GAP-ES consists of 30 items, the minimum required sample size would be 150 participants. Our sample size exceeds this minimum requirement, enhancing the reliability of the findings.

### 2.2. Instrument Translation and Cultural Adaptation

The adaptation of the GAP Scale into Spanish followed the standardized guidelines for cross-cultural adaptation of self-report measures as proposed by Beaton et al. [20]. The process involved several steps to ensure linguistic and conceptual equivalence between the original English version and the Spanish translation. Initially, two independent bilingual translators whose native language is Spanish translated the original GAP Scale into Spanish. Both translators were knowledgeable about the subject matter but worked independently to prevent bias. The translations were then synthesized into a single version after resolving discrepancies through discussion. Next, the synthesized Spanish version was back-translated into English by two different bilingual translators who were blinded to the original GAP Scale. These translators were native English speakers with proficiency in Spanish but had no prior exposure to the instrument. The purpose of the back-translation was to identify any inconsistencies or inaccuracies in the translation process. An expert committee consisting of healthcare professionals and academics with expertise in LGBT healthcare, public health, psychology, and translation methodology reviewed all versions of the questionnaire. An expert committee consisting of healthcare professionals and academics with expertise in LGBT healthcare, public health, psychology, and translation methodology reviewed all versions of the questionnaire. The committee included a health promotion specialist, a public health specialist, a paramedic, dietitians, a nurse, a psychologist, and a physician, some of whom had specific knowledge of LGBT cultural dynamics within Spain. In adapting the instrument for a Spanish-speaking audience, particular attention was given to cultural nuances relevant to LGBT healthcare. This included careful consideration of language and phrasing to ensure cultural appropriateness and sensitivity within the Spanish context, acknowledging linguistic and societal factors unique to Spanish-speaking populations. This input was instrumental in aligning the instrument with the specific cultural and social context of the target population. The committee compared the original and back-translated versions to ensure conceptual equivalence and cultural relevance, making adjustments where necessary. To assess the clarity and comprehensibility of the GAP-ES, cognitive interviews were conducted with a sample of 20 volunteers from the target population. The primary purpose of these interviews was to identify any linguistic ambiguities, cultural misinterpretations, or items that could cause confusion among respondents. The feedback allowed for minor adjustments to the wording and phrasing of certain items, ensuring that the instrument would be easily understood and applicable within the Spanish context. This pre-testing phase helped minimize potential misunderstandings and enhanced the validity of the questionnaire for the main study.

### 2.3. Description of the Gay Affirmative Practice Scale

The Gay Affirmative Practice Scale (GAP) is a psychometric instrument developed by Catherine Crisp to assess clinicians’ beliefs and behaviors regarding their work with lesbian, gay, bisexual, and transgender (LGBT) clients [17]. The scale aims to measure affirmative practices and attitudes, promoting a supportive and respectful approach toward LGBT individuals within healthcare settings. The GAP Scale consists of 30 items divided into two subscales: Beliefs (items 1–15) and Behaviors (items 16–30). The Beliefs subscale assesses clinicians’ attitudes and beliefs about LGBT individuals and issues. Respondents indicate their level of agreement with statements using a 5-point Likert scale ranging from “strongly disagree” to “strongly agree”. Sample items include statements like “Practitioners should educate themselves about gay and lesbian lifestyles” and “Practitioners should take advantage of professional development opportunities to improve their practice with gay and lesbian clients”. The Behaviors subscale evaluates the frequency of affirmative practices when working with LGBT clients. Participants respond using a 5-point scale ranging from “never” to “always”. Examples of items include “I inform clients about gay affirmative resources in the community” and “I show comfort with gay and lesbian issues to gay and lesbian clients”.

Higher scores on the GAP Scale indicate a more affirmative practice toward LGBT clients. The original English version demonstrated high internal consistency, with Cronbach’s alpha coefficients of 0.93 for the Beliefs subscale and 0.94 for the Behaviors subscale [17].

### 2.4. Statistical Analysis

Statistical analyses were performed using R software version 4.2.2 and RStudio GUI [21], utilizing packages such as “psy”, “lavaan”, “psych”, and “diagram” [22,23,24,25,26]. There were no missing data, as participants were required to complete the entire questionnaire. Descriptive statistics were calculated for demographic variables. The internal consistency of the GAP-ES was assessed using Cronbach’s alpha coefficient. The following thresholds were applied: α ≥ 0.9 (excellent), 0.8 ≤ α < 0.9 (good), 0.7 ≤ α < 0.8 (acceptable), 0.6 ≤ α < 0.7 (questionable), 0.5 ≤ α < 0.6 (poor), and α < 0.5 (unacceptable). Confirmatory Factor Analysis (CFA) was conducted to evaluate the factor structure of the GAP-ES. The Diagonally Weighted Least Squares (DWLS) method was used due to the ordinal nature of the data, making it an ideal choice for Likert-scale responses, as it provides robust estimates without assuming multivariate normality. This ensures accurate parameter estimates suited to the psychometric analysis of the instrument. Model fit was assessed using the Standardized Root Mean Square Residual (SRMR), Root Mean Square Error of Approximation (RMSEA), Comparative Fit Index (CFI), and Tucker–Lewis Index (TLI). The Hu and Bentler two-index strategy was applied, and it was considered a good fit when SRMR < 0.09 and at least one of the following applied: CFI > 0.96, TLI > 0.96, or RMSEA < 0.06 [27]. Item analysis included calculating item-total correlations and examining the discriminative power of each item. Statistical significance was set at *p* < 0.05 for all analyses.

## 3. Results

### 3.1. Study Population

The study included 236 participants, of which the majority were women (64.41%), while men constituted 35.59%. Participants were distributed across various age groups ranging from 18 to 63 years, with the most common age bracket being 39 years (IQR: 39–46). Most participants lived in large cities, with 42.80% residing in cities with over 500,000 inhabitants. Regarding sexual orientation, 58.05% of the participants identified as heterosexual, 28.81% as homosexual, and 13.14% as bisexual.

In terms of relationship status, 38.56% were married, and 34.75% were in a formal partnership, while 19.49% were single. The majority of participants were nurses or other healthcare-related specialists (72.88%), with physicians making up 9.32% of the sample. Detailed information on other healthcare professions represented in the sample can be found in Table 1. When it comes to LGBT-related training in the last five years, 61.44% of participants reported having never attended any such training, while 29.66% had attended one to two sessions (Table 1).

**Figure 1:**
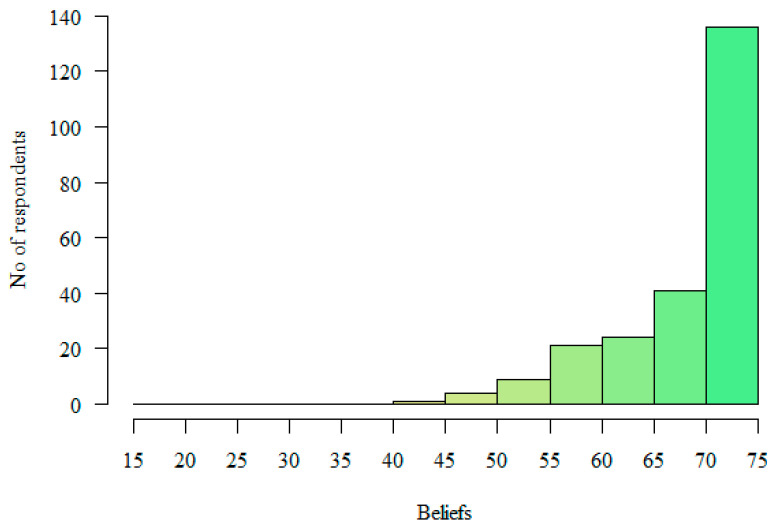
Distribution of scores on the “Beliefs” subscale.

**Figure 2:**
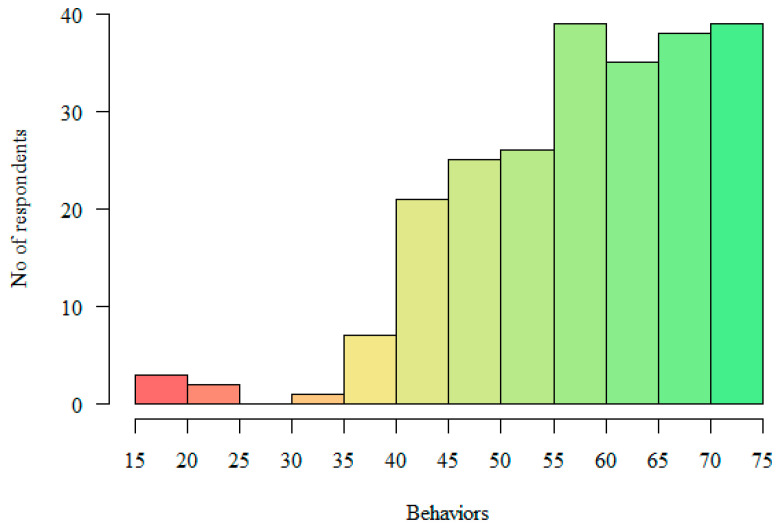
Distribution of scores on the “Behaviors” subscale.

The GAP Scale questionnaire evaluates respondents’ beliefs and behaviors concerning their approach to LGBT patients/clients, with both scales scored between 15 and 75 points. A higher score signifies a more affirmative stance. The GAP Scale does not specify a set point range to qualify as affirmative. In this study, respondents scored an average of 69.14 points (SD = 7.13) on the Beliefs scale. Meanwhile, the mean score on the Behaviors scale was 58.47 points (SD = 11.84). See Table 2 and Figure 1 and Figure 2 for further details. The colors used in Figure 1 and Figure 2 represent different levels of respondent scores. Green indicates the highest levels, while red represents the lowest levels. This color scheme allows for a quick comparison of the distribution of various behaviors or characteristics across a range of values.

### 3.2. Analysis of Individual Items

Table 3 presents the analysis results for each individual questionnaire item. A significant ceiling effect was observed in items 1, 2, 4, and 23. It is important to note that ‘missing data’ in this context refers to the absence of a floor or ceiling effect in specific items rather than incomplete responses from participants, as all participants provided complete answers across all items in the questionnaire.

### 3.3. Tool Reliability

#### 3.3.1. Internal Consistency

Since the GAP items are expressed on an ordinal scale rather than a continuous one, the Diagonally Weighted Least Squares method was employed. The original GAP structure is two-factorial, comprising beliefs (Items 1–15) and behaviors (Items 16–30). For this structure, satisfactory fit indices were obtained, as detailed in Table 4.

**Figure 3:**
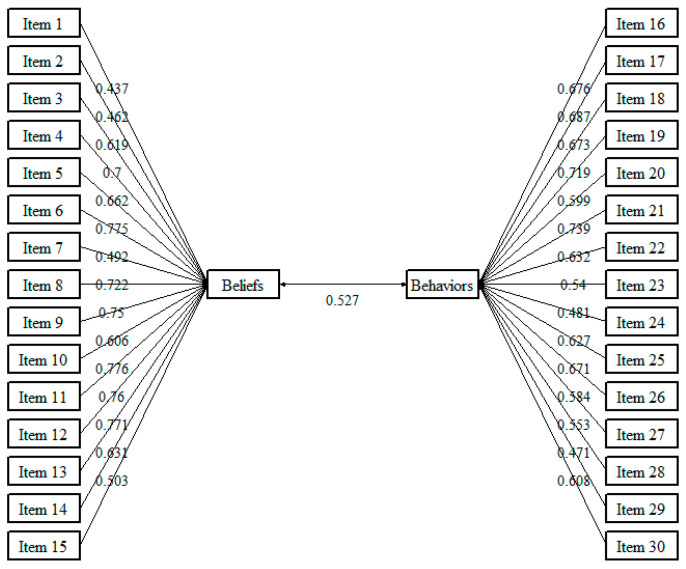
Path diagram for CFA of the GAP-ES.

The factor loadings of the individual items ranged from 0.437 to 0.776 and were statistically significant (*p* < 0.05)—see Table 5. Figure 3 shows the path diagram for CFA of the GAP-ES.

#### 3.3.2. Cronbach’s Alpha

Cronbach’s alpha values for the subscales are presented in Table 6. The Beliefs subscale has an alpha of 0.915, and the Behaviors subscale has an alpha of 0.902, indicating that both subscales demonstrate high reliability.

Cronbach’s alpha values for the Beliefs subscale are presented in Table 7. The internal consistency of this subscale remains high, with a slight increase in alpha observed when item 1 is excluded, raising the alpha from 0.915 to 0.916. This marginal change is not significant enough to warrant the removal of the item. Similarly, for the Behaviors subscale, no exclusion of any single item results in a meaningful improvement of Cronbach’s alpha, as it remains consistent at around 0.902, further demonstrating the reliability of both subscales.

#### 3.3.3. McDonald’s Omega

The value of McDonald’s Omega (ω) is 0.942, indicating a high level of reliability for the scales as well.

## 4. Discussion

The psychometric assessment of the Spanish-language Gay Affirmative Practice Scale (GAP-ES) centered on evaluating its internal consistency, reliability, and factor structure. The results confirmed that the GAP-ES is a valid and reliable instrument that is effectively adapted to the Spanish-speaking context while maintaining strong psychometric properties akin to the original GAP scale. This adaptation is particularly significant, as it represents the inaugural effort to translate and validate the GAP scale for Spanish-speaking healthcare professionals, thereby enhancing the evaluation of affirmative beliefs and behaviors within this demographic.

In terms of internal consistency, the GAP-ES demonstrated high reliability, with Cronbach’s alpha coefficients of 0.91 for the Beliefs subscale and 0.90 for the Behaviors subscale. These figures are closely aligned with those reported in the original English version by Crisp et al., which were 0.93 and 0.94 for the Beliefs and Behaviors subscales, respectively [17]. This consistency underscores the reliability of the GAP-ES in measuring affirmative practices among healthcare professionals in Spanish-speaking settings. When comparing the GAP-ES with other language adaptations like the Polish version (GAP-PL), all versions exhibit robust internal consistency and reliability [28]. The GAP-PL reported Cronbach’s alpha values of 0.915 for the Beliefs subscale and 0.902 for the Behaviors subscale, mirroring the strong psychometric properties observed in both the original and Spanish versions [28]. Additionally, the ceiling effect observed in items 1, 2, 4, and 23 suggests that these statements may represent widely accepted principles among healthcare professionals, resulting in high levels of agreement. This could indicate that these items are relatively easy or not sufficiently discriminative in assessing subtle variations in affirmative practices. In future iterations of the GAP-ES, refining these items to capture more specific or nuanced aspects of LGBT-affirmative care may enhance the scale’s ability to differentiate levels of agreement among respondents.

The ability to properly measure and assess the level to which professionals and care providers are able to demonstrate affirming and supportive practice to LGBT patients and communities is a critical component of addressing and reducing the disparities experienced by LGBT individuals. Affirming practice models provide the kinds of guidelines, behaviors, and beliefs needed to provide care that is not only sound but culturally competent and supportive as well. As stated by Davies, “[gay affirmative practice] affirms a lesbian, gay, or bisexual identity as an equally positive human experience and expression to heterosexual identity” [29]. This is a reminder that the absence of outright hostile or homophobic attitudes or behaviors towards LGBT people is not adequate to be able to practice in an affirming way. Instead, affirmation is the ongoing and purposeful work to validate, recognize, honor, and value the identities of LGBT people [30]. Those practitioners who have been shown to actively be engaging in inclusive and affirming practices in the course of their work are known to be actively reducing health disparities for LGBT populations across a number of chronic illnesses (including cancer and HIV testing) and even in areas that may not be initially expected, such as annual influenza vaccination [5].

Having a tool such as the GAP-ES allows for this kind of assessment and evaluation to be conducted across different language and cultural groups that practice within the field of health care. The data and findings from tools like the GAP-ES allow for potential areas of deficiency to be identified so that education and training at both the pre-professional and post-professional levels can be tailored to the needs of the practitioners within that cultural group. There continues to be a greater call for health science curricula and other educational preparation programs in the healthcare field to include subject matter related to LGBT health and gender-affirming health. This is due to the fact that providers routinely indicate that they are not adequately prepared by their current educational and professional training programs, as well as the recognition that there is an ethical imperative to bring these conversations into all levels of preparation and training for providers [31]. As many patients are still nervous about disclosing their sexual orientation and/or gender identity to their care providers, critical details and information related to their health needs and disease risks can often go unreported, which makes it harder for providers to know the kinds of unique care needs and resources that may be needed [32]. By having a tool that is culturally and linguistically tailored to a particular population and community, current levels of providers’ strengths and deficiencies can be identified early and addressed as needed in order to further enhance the effectiveness and affirming nature of how providers practice. Additionally, the demographic diversity of our sample, including factors such as gender, age, and professional background, may provide meaningful insights into the understanding of affirmative practices toward LGBT patients. Previous research suggests that these demographic characteristics can influence healthcare professionals’ comfort and competency in delivering LGBT-affirming care [33,34]. Furthermore, the finding that 61.44% of participants had not attended LGBT-related training underscores a critical gap in professional preparation that may impact the efficacy of affirmative practices. Recognizing how these elements, alongside the limited training, relate to the beliefs and behaviors observed in this study highlights potential areas for targeted education and support across various professional groups, further enhancing the practical application of the GAP-ES tool and setting the stage for future interventions.

The GAP-ES also has potential applications in structured training programs. For instance, it could be integrated into pre-professional training for students in medical, nursing, and allied health fields, helping to cultivate LGBT-affirmative competencies before entering the workforce. In post-professional settings, the tool could be used as part of continuing education programs, providing healthcare providers with ongoing feedback on their affirmative practices and identifying areas where additional training may be beneficial. By incorporating the GAP-ES into both pre- and post-professional training, institutions can foster a more inclusive healthcare environment that is responsive to the needs of LGBT patients.

Lastly, there are additional practical considerations and implications for this tool that are valuable to recognize as well. It has been documented that the political environments and the societal beliefs and attitudes in which a practitioner lives, learns, and works can have measurable impacts on how they interact with patients, as well as the kinds of care plans that are or are not developed and provided [35,36]. As such, it cannot be assumed that healthcare professionals are immune to the ways in which the societal circumstances in which they find themselves can influence how they practice, especially at the implicit and unconscious levels [37]. Not only does this show the need for tools like the GAP Scale to exist in order to regularly and consistently assess the beliefs and attitudes of professionals, but it also shows the need for this scale to be properly adapted to and available for use in a multitude of different languages. The more such scales can be translated and shown to be valid for use in different linguistic and cultural groups, the greater the insight that can be gained into how those practitioners who belong to and practice within different linguistic and cultural groups are influenced by their language and culture regarding how they interact with and treat LGBT patients and communities.

### Study Limitations

There are several important limitations that should be considered in this study. Although the sample size was sufficient for the primary research objectives, increasing the number of participants could yield a more comprehensive understanding of the issues examined. The use of a convenience sampling method through online platforms, such as Instagram and Facebook, may have introduced selection bias, potentially excluding individuals without consistent or reliable internet access, as well as those less active online. This limits the diversity of the sample and could reduce the generalizability of the findings to populations with less access to digital technologies. Additionally, the sample may not fully reflect the general demographics of healthcare professionals in the region, as the use of social media platforms like Instagram and Facebook might exclude individuals who are less active online. Furthermore, we observed a ceiling effect in several items (e.g., items 1, 2, 4, and 23), where a high percentage of respondents provided the most affirmative responses. This pattern may reflect a strong, widespread endorsement of LGBT-affirmative attitudes and behaviors among healthcare professionals, indicating that these items tap into universally accepted principles within the sample. While this suggests that the GAP-ES is effective in capturing general affirmative beliefs, future research could explore whether more nuanced or specific statements might help to further differentiate levels of LGBT-affirmative practices. Lastly, while the psychometric properties of the translated instrument were assessed, further research should explore its application in different cultural contexts to ensure broad validity and reliability.

## 5. Conclusions

The findings from the Spanish adaptation of the Gay Affirmative Practice Scale (GAP-ES) indicate that the instrument demonstrates strong internal consistency, reliability, and an appropriate factor structure. These outcomes suggest that the GAP-ES is a robust tool for evaluating affirmative practices among healthcare professionals in Spanish-speaking regions. Additionally, the psychometric analysis and cultural adaptation process produced results that are comparable to those of the original English version, confirming that the GAP-ES can be effectively applied for both research and clinical assessments within the healthcare sector. Beyond clinical settings, the GAP-ES offers valuable potential for broader scientific research and professional development programs aimed at strengthening LGBT-affirmative competencies. By identifying areas for improvement, the tool can guide targeted training interventions and foster a more inclusive, culturally competent healthcare environment. Its adaptability also makes it suitable for investigating affirmative practices across diverse contexts and populations, contributing to a deeper understanding of LGBT-affirmative care on a global scale.

## Figures and Tables

**Table 1:** Demographic and professional characteristics of the study population.

Parameter		Total (N = 236)
Gender	Man	84 (35.59%)
	Woman	152 (64.41%)
Age [years]	Mean (SD)	38.27 (9.76)
	Median (quartiles)	39 (31–46)
	Range	18–63
	n	236
Place of residence	Village	33 (13.98%)
	City: up to 20,000 inhabitants	6 (2.54%)
	City: 20,000–100,000 inhabitants	41 (17.37%)
	City: 100,000–500,000 inhabitants	55 (23.31%)
	City: more than 500,000 inhabitants	101 (42.80%)
Sexual orientation	Heterosexual	137 (58.05%)
	Homosexual	68 (28.81%)
	Bisexual	31 (13.14%)
Relationship status	Single	46 (19.49%)
	In marriage	91 (38.56%)
	In a (formal) partnership	82 (34.75%)
	In a non-formalized relationship	5 (2.12%)
	Divorce/Separation	10 (4.24%)
	Widower/Widow	2 (0.85%)
Profession	Nurses and their specialties	172 (72.88%)
	Doctors	22 (9.32%)
	Clinical psychologist	5 (2.12%)
	Physiotherapists	4 (1.69%)
	Pharmacists	2 (0.85%)
	Podiatrists	2 (0.85%)
	Dentists	1 (0.42%)
	Occupational therapists	1 (0.42%)
	Student	12 (5.08%)
	Other health profession	15 (6.36%)
How many training courses (conferences, webinars) have you attended in the last 5 years that dealt in any way with LGBT patients?	Never	145 (61.44%)
	1–2 times	70 (29.66%)
	3–5 times	14 (5.93%)
	More than 5 times	7 (2.97%)

**Table 2:** Subscale results.

GAP	Point Range	N	Mean	SD	Median	Min	Max	Q1	Q3
Beliefs	15–75	236	69.14	7.13	72	43	75	65.75	75
Behaviors	15–75	236	58.47	11.84	59.5	15	75	50.75	68

**Table 3:** Detailed analysis of individual items within the questionnaire.

Item	Floor Effect	Ceiling Effect	Missing Data
1	0.4%	88.1%	0.0%
2	0.0%	86.9%	0.0%
3	0.8%	72.9%	0.0%
4	0.0%	82.2%	0.0%
5	0.4%	53.0%	0.0%
6	0.8%	74.2%	0.0%
7	2.1%	74.2%	0.0%
8	0.0%	69.1%	0.0%
9	0.8%	57.2%	0.0%
10	0.0%	58.9%	0.0%
11	0.4%	71.2%	0.0%
12	0.8%	70.3%	0.0%
13	0.0%	70.8%	0.0%
14	0.4%	81.8%	0.0%
15	0.0%	80.5%	0.0%
16	5.1%	55.9%	0.0%
17	5.1%	53.0%	0.0%
18	9.3%	34.3%	0.4%
19	17.4%	28.0%	0.0%
20	8.9%	40.7%	0.0%
21	17.8%	35.6%	0.0%
22	13.6%	40.3%	0.0%
23	2.1%	84.7%	0.0%
24	2.5%	37.3%	0.0%
25	13.1%	18.2%	0.0%
26	5.1%	31.8%	0.0%
27	1.3%	80.1%	0.0%
28	2.5%	72.5%	0.0%
29	5.5%	74.2%	0.0%
30	4.2%	50.8%	0.0%

**Table 4:** Results of fit indices.

Chi-Square Test			RMSEA	CFI	TLI	SRMR
χ^2^	df	*p*				
330.96	404	0.997	<0.001	>0.999	>0.999	0.071

**Table 5:** The analysis of the internal consistency of the GAP-ES.

Domain	Item	Loading	*p*
Beliefs	1	0.437	*p* < 0.001
	2	0.462	*p* < 0.001
	3	0.619	*p* < 0.001
	4	0.700	*p* < 0.001
	5	0.662	*p* < 0.001
	6	0.775	*p* < 0.001
	7	0.492	*p* < 0.001
	8	0.722	*p* < 0.001
	9	0.750	*p* < 0.001
	10	0.606	*p* < 0.001
	11	0.776	*p* < 0.001
	12	0.760	*p* < 0.001
	13	0.771	*p* < 0.001
	14	0.631	*p* < 0.001
	15	0.503	*p* < 0.001
Behaviors	16	0.676	*p* < 0.001
	17	0.687	*p* < 0.001
	18	0.673	*p* < 0.001
	19	0.719	*p* < 0.001
	20	0.599	*p* < 0.001
	21	0.739	*p* < 0.001
	22	0.632	*p* < 0.001
	23	0.540	*p* < 0.001
	24	0.481	*p* < 0.001
	25	0.627	*p* < 0.001
	26	0.671	*p* < 0.001
	27	0.584	*p* < 0.001
	28	0.553	*p* < 0.001
	29	0.471	*p* < 0.001
	30	0.608	*p* < 0.001

**Table 6:** Cronbach’s alpha values for instrument subscales.

Domain	Cronbach’s Alpha
Beliefs	0.915
Behaviors	0.902

**Table 7:** Cronbach’s alpha values after excluding individual items.

Domain	Item	Alpha with Item Omitted	Domain	Item	Alpha with Item Omitted
Beliefs	1	0.916	Behaviors	16	0.891
	2	0.913		17	0.89
	3	0.907		18	0.892
	4	0.908		19	0.893
	5	0.91		20	0.897
	6	0.908		21	0.891
	7	0.915		22	0.894
	8	0.908		23	0.9
	9	0.908		24	0.899
	10	0.911		25	0.895
	11	0.906		26	0.894
	12	0.905		27	0.899
	13	0.905		28	0.897
	14	0.911		29	0.901
	15	0.914		30	0.897

## Data Availability

The data will be accessible upon request from the primary author.

## Supplementary Materials

The following supporting information can be downloaded at: https://www.mdpi.com/article/10.3390/healthcare12222258/s1, The Spanish Version of the Gay Affirmative Practice Scale (GAP-ES)**.**
