# Supplementary material for: Cultural Adaptation and Psychometric Properties of the Spanish Version of the Gay Affirmative Practice Scale (GAP-ES)
Source: Healthcare (Basel). 2024 Nov 13;12(22):2258. doi: 10.3390/healthcare12222258 (PMC11593571; doi:10.3390/healthcare12222258)
Supplement: Supplementary file 1 [file healthcare-12-02258-s001.zip › healthcare-3278952-supplementary.pdf]

ESCALA DE PRÁCTICAS AFIRMATIVAS HOMOSEXUALES – Versión Española (GAP-ES)
The Spanish Version of the Gay Affirmative Practice Scale (GAP-ES)

Este cuestionario está diseñado para medir las creencias del personal sanitario sobre el tratamiento de pacientes gays y lesbianas y su comportamiento en el ámbito clínico con estos pacientes. No hay respuestas correctas o incorrectas.
Responda a cada pregunta con la mayor sinceridad posible.
Por favor, valore en qué medida está de acuerdo o en desacuerdo con cada una de las afirmaciones sobre el trato a los clientes gays o lesbianas según la escala que aparece a continuación:

SA = Totalmente de acuerdo (Strongly agree)
A = De acuerdo (Agree)
N = Ni de acuerdo ni en desacuerdo (Neither agree nor disagree)
D = En desacuerdo (Disagree)
SD = Totalmente en desacuerdo (Strongly Disagree)

1. En su práctica con pacientes gays/lesbianas, los profesionales sanitarios deben apoyar la diversidad de sus familias. ....
2. Los profesionales sanitarios deben manifestar su respeto por el estilo de vida gay/lesbiana. ....
3. Los profesionales sanitarios deben hacer un esfuerzo por conocer la diversidad dentro de la comunidad gay/lesbiana. ....
4. Los profesionales sanitarios deben conocer los recursos existentes y las oportunidades para que los gays/lesbianas se beneficien de la atención médica. ....
5. Los profesionales sanitarios deben informarse sobre el estilo de vida gay/lésbico. ....
6. Los profesionales sanitarios deben ayudar a los pacientes gays/lesbianas a desarrollar una identidad positiva como personas gays/lesbianas. ....
7. Los profesionales sanitarios deben cuestionar la información errónea sobre los pacientes gays/lesbianas. ....
8. Los profesionales sanitarios deben aprovechar las oportunidades de desarrollo profesional para mejorar su práctica cuando trabajan con pacientes gays/lesbianas. ....
9. Los profesionales sanitarios deben animar a los pacientes gays/lesbianas a formar redes que apoyen su orientación. ....
10. Los profesionales sanitarios deben conocer los problemas específicos de las parejas gays y lesbianas. ....
11. Los profesionales sanitarios deben adquirir los conocimientos necesarios para trabajar eficazmente con pacientes gays/lesbianas. ....
12. Los profesionales sanitarios deben trabajar para desarrollar las habilidades necesarias para trabajar eficazmente con pacientes gays/lesbianas. ....
13. Los profesionales sanitarios deben trabajar en el desarrollo de sus propias actitudes necesarias para trabajar eficazmente con pacientes gays/lesbianas. ....
14. Los profesionales sanitarios deben ayudar a los pacientes a reducir la vergüenza asociada a los sentimientos homosexuales. ....
15. La discriminación crea problemas que los pacientes gays/lesbianas pueden necesitar abordar en terapia. ....

Por favor, valore la frecuencia con la que realiza cada uno de los comportamientos con pacientes gays y lesbianas basándose en la siguiente escala:

A= Siempre (Always)
U= Típicamente (Usualy)
S= A veces (Sometimes)
R= Raro (Rarely)
N= Nunca (Never)

16. Ayudo a los pacientes a reducir la vergüenza asociada a los sentimientos homosexuales ....
17. Ayudo a los pacientes gays y lesbianas a afrontar los problemas derivados de los prejuicios sociales. ....
18. Informo a los pacientes sobre la posibilidad de recibir apoyo dedicado a su orientación en la sociedad ....
19. Conciencio a los pacientes de las consecuencias de vivir en una sociedad homófoba ....
20. Respondo a la orientación sexual del paciente cuando es necesario para la terapia ....
21. Ayudo a gays y lesbianas a superar la presión religiosa que han sufrido a causa de su orientación sexual. ....
22. Dirijo interacciones que aumentan la seguridad de los pacientes gays y lesbianas ....
23. Señalo que la orientación homosexual es tan sana como la heterosexual. ....
24. Conozco bien los temas relacionados con gays y lesbianas y puedo hablar de ellos. ....
25. Ayudo a los pacientes a reconocer su homofobia interiorizada ....
26. Mejoro constantemente mi propia competencia en cuestiones relacionadas con los problemas de los pacientes gays/lesbianas. ....
27. Tengo una mentalidad abierta a la hora de adaptar la terapia a las necesidades específicas de las personas gays/lesbianas. ....
28. Creo un clima que permita la autoidentificación voluntaria de gays y lesbianas ....
29. Hablo con los pacientes sobre su orientación sexual de forma no amenazadora. ....
30. Facilito que los gays y lesbianas expresen su rabia por la opresión que han sufrido. ....

Guía de puntuación: Utilizando la tabla siguiente, atribuya a cada respuesta el número de puntos indicado. Después de responder a todas las preguntas, suma la puntuación total. Las puntuaciones más altas reflejan una práctica más afirmativa con gays y lesbianas.

Preguntas 1 a 15	Preguntas 16-30	Puntos
Totalmente de acuerdo	Siempre	5
Estoy de acuerdo	Normalmente	4
Ni de acuerdo ni en desacuerdo (no tengo opinión)	A veces	3
No estoy de acuerdo	Raramente	2
Estoy totalmente en desacuerdo	Nunca	1

Karniej, P.; Dissen, A.; Juárez-Vela, R.; Martinez Sabater, A.; Pozo-Herce, P.d.; Gea-Caballero, V.; Echaniz-Serrano, E.; Czapla, M. Cultural Adaptation and Psychometric Properties of the Spanish Version of the Gay Affirmative Practice Scale (GAP-ES). *Healthcare* **2024**, *12*, 2258. https://doi.org/10.3390/healthcare12222258
